# Rural–Urban Differences in Overweight and Obesity, Physical Activity, and Food Security Among Children and Adolescents

**DOI:** 10.5888/pcd20.230136

**Published:** 2023-10-19

**Authors:** Elizabeth Crouch, Demetrius A. Abshire, Michael D. Wirth, Peiyin Hung, Gabriel A. Benavidez

**Affiliations:** 1 Rural and Minority Health Research Center, Arnold School of Public Health, University of South Carolina, Columbia; 2 Department of Health Services Policy and Management, Arnold School of Public Health, University of South Carolina, Columbia; 3 Biobehavioral Health and Nursing Science, College of Nursing, University of South Carolina, Columbia

## Abstract

**Introduction:**

Childhood obesity has been associated with numerous poor health conditions, with geographic disparities demonstrated. Limited research has examined the association between rurality and food security, physical activity, and overweight or obesity among children. We examined rates of food security, physical inactivity, and overweight or obesity among rural and urban children and adolescents, and associations between rurality and these 3 outcomes.

**Methods:**

We used cross-sectional data from a nationally representative sample of children and adolescents aged 10 to 17 years from the 2019–2020 National Survey of Children’s Health (N = 23,199). We calculated frequencies, proportions, and unadjusted associations for each variable by using descriptive statistics and bivariate analyses. We used multivariable logistic regression models to examine the association between rurality and food security, physical activity, and overweight or obesity.

**Results:**

After adjusting for sociodemographic factors, rural children and adolescents had higher odds than urban children and adolescents of being overweight or obese (adjusted odds ratio = 1.30; 95% CI, 1.11–1.52); associations between rurality and physical inactivity and food insecurity were not significant.

**Conclusion:**

The information from this study is timely for policy makers and community partners to make informed decisions on the allocation of healthy weight and obesity prevention programs for children and adolescents in rural settings. Our study provides information for public health programming and the designing of appropriate dietary and physical activity interventions needed to reduce disparities in obesity prevention among children and adolescents.

**Summary**

**What is already known on this topic?**

Childhood obesity is associated with numerous poor health conditions, with geographic disparities demonstrated. Limited research has examined the association between rurality and food security, physical activity, and overweight or obesity among children.

**What is added by this report?**

We examined rates of food security, physical inactivity, and overweight or obesity among rural and urban children and associations between rurality and these 3 outcomes. These data are needed to inform public health programming and to design appropriate dietary and physical activity interventions.

**What are the implications for public health practice?**

Our findings highlight the need for further research examining drivers of obesity disparities among children in rural communities.

## Introduction

Nearly 20% of children and adolescents in the US are obese and 16% are overweight (1). Childhood obesity is associated with multiple poor health conditions, including diabetes, sleep apnea, cardiovascular disease, and others (2,3). Children and adolescents who are overweight or obese are more likely to remain overweight or obese into adulthood (4). Demographic differences in overweight and obesity among children and adolescents have been demonstrated, with Hispanic children more likely to be overweight or obese than non-Hispanic children (5). Geographic differences in obesity have also been demonstrated among rural and urban children and adolescents, with rural children and adolescents having a higher likelihood of obesity than their urban counterparts (6,7). Research shows an inverted relationship between severe obesity and urbanization, with severe obesity highest in more rural areas (8).

Rural–urban differences in access to nutrition and physical activity may influence rates of overweight and obesity among children and adolescents (9), but the evidence that food environments are associated with health outcomes among children and adolescents is limited (10). Although rural areas are more likely than urban areas to be food deserts, rural–urban differences in food insecurity are more nuanced (11). The prevalence of food insecurity has been reported to be approximately 11% in rural areas, lowest in suburban areas (8.8%), and highest in cities (12.2%) (12). These findings may be related to differences in poverty by rurality. Poverty rates in rural areas are higher than in urban areas, and are increasing faster in rural areas than in urban areas (13).

Research has had conflicting results on differences in physical activity levels between rural and urban children and adolescents, with some studies showing urban children and adolescents were less active than rural children and adolescents, and others showing that rural children and adolescents were more involved in sports teams (14,15). Other research showed that children living in urban areas are the least active but that children in small cities are slightly more active than urban children (16). One reason for these mixed findings may be the differing built environments across the rural–urban continuum (17). Barriers to physical activity in rural areas include traffic, safety, and lack of sidewalks, gyms, and parks (17). However, children living in rural areas may have more opportunities to engage in physical activity in the natural environment, which is a finding echoed among adults living in rural areas (18).

The Healthy People 2020 objectives included reducing disparities in childhood obesity (19). Few studies have used a national sample to examine rural–urban differences in overweight and obesity, as well as physical activity, among children and adolescents (20,21). These studies are now dated and did not include food security. An updated study is needed for informing public health programming and designing appropriate dietary and physical activity interventions.

The objectives of this study were 1) to examine rates of food security, physical inactivity, and overweight or obesity among rural and urban children and adolescents and 2) to examine the association between rurality and these 3 outcomes among a national sample of children and adolescents aged 10 to 17 years. We hypothesized that rural children and adolescents, compared with their urban counterparts, would be less likely to be food secure, less likely to engage in appropriate levels of physical activity, and more likely to be overweight or obese. Findings from this study will be informative for reducing disparities in obesity prevention among children and adolescents.

## Methods

Data for this study were from the National Survey of Children’s Health (NSCH) in 2019 and 2020. The NSCH is a cross-sectional, nationally representative dataset of US children and adolescents (hereinafter, children). The survey is administered by the NSCH to adult (aged ≥18 y) participants by mail and online (childhealthdata.org). To participate in the study, the participant must be a caregiver of a child aged 0 to 17 years; 1 child in the household is randomly chosen by the NSCH study software, and the adult participant responds to questions about that child. The 2019 survey was completed before the pandemic, and the 2020 survey was completed during the middle of the pandemic (July 2020 through January 2021). The NSCH in 2019 and 2020 (hereinafter, NSCH 2019-2020) had a total of 72,210 completed interviews, with results weighted by the population of US children.

We restricted our study to children aged 10 years or older, because the NSCH suppresses information on weight for children younger than 10 years. This restriction resulted in a sample of children aged 10 to 17 with a known body mass index (BMI, calculated as weight in kilograms divided by height in meters squared [kg/m^2^]; n = 37,774). The sample was further restricted to children whose rural or urban residence was identified in the public use dataset. The NSCH does not release information on residence for states whose population of children is less than 100,000. The following states did not have data on residence in the NSCH 2019–2020: Alaska, Delaware, Hawaii, Maine, Mississippi, Montana, New Hampshire, North Dakota, Rhode Island, South Dakota, Vermont, West Virginia, and Wyoming. This restriction resulted in a final sample (n = 23,199) representing 37 states and the District of Columbia.

The NSCH includes 4 geographic variables: state of residence, core-based statistical area, metropolitan statistical area, and metropolitan principal city status. Children who did not live in a metropolitan statistical area were considered rural, on the basis of the Office of Management and Budget definition, which defines populations of 50,000 individuals or more as residing in a metropolitan statistical area and those with less than 50,000 people as nonmetropolitan, or rural, for this study.

BMI in the NSCH is measured by using the caregiver’s recollection of the child’s height and weight. BMI is calculated by NSCH for children aged 10 to 17 years as 1) underweight (BMI <5th percentile, 2) healthy weight (5th percentile to <85th percentile, and 3) overweight or obese (≥85th percentile). These categories were established by the Centers for Disease Control and Prevention (22).

For physical activity, the survey asked, “During the past week, on how many days did this child exercise, play a sport, or participate in physical activity for at least 60 minutes?” Response options included 0 days, 1 to 3 days, 4 to 6 days, and every day. Responses of less than every day were considered physically inactive. The US Department of Health and Human Services’ physical activity guidelines recommend that children have at least 60 minutes of physical activity each day (23).

For food insecurity, the survey asked, “Which of these statements best describes your household’s ability to afford the food you need during the past 12 months?” Response options were the following: We could always afford to eat good nutritious meals; We could always afford enough to eat but not always the kinds of food we should eat; Sometimes we could not afford enough to eat; Often we could not afford enough to eat. We categorized respondents who reported that they could always afford enough to eat, but not always the kinds of food we should eat, as experiencing mild food insecurity. We categorized those who responded that they could sometimes or often not afford enough to eat as experiencing moderate to severe food insecurity. Respondents who reported they could always afford good, nutritious meals were categorized as being food secure. For the regression analysis, we further combined these categories into food secure and mild-severe food insecurity (24).

To model health care access and use, we used the Andersen Behavioral Model of Health Services Use (25), which draws on the theory of predisposing, enabling, and need characteristics, as well as health behaviors. Thus, we selected covariates a priori. For predisposing characteristics, we included the sex, age, and race and ethnicity of the child. We classified age into 3 categories: 10 to 12 years, 13 to 15 years, and 16 to 17 years. We classified race and ethnicity as Hispanic, non-Hispanic Black, non-Hispanic White, and non-Hispanic multiracial or other (includes non-Hispanic Asian, American Indian or Alaska Native, Native Hawaiian or other Pacific Islander). Enabling characteristics were caregiver and household characteristics: primary language spoken in the household, the highest level of educational attainment of a caregiver in the household, family structure, and household income as a percentage of the federal poverty level (FPL). We categorized primary language spoken in the household as English or not English. We categorized educational attainment as less than or equal to a high school diploma/GED (General Educational Development) or some college or more. Categories of family structure were 2 parents, currently married; 2 parents, not currently married; single caregiver, and other. Household income as a percentage of the FPL, categorized as 0% to 99%, 100% to 199%, 200% to 399%, and 400% or above.

We used the NSCH special health care needs tool to determine if the child had special health care needs. This tool asks about the use of prescription medication, functional limitations, elevated use of services, or specialized therapy, and ongoing developmental, emotional, or behavioral conditions. A child was defined as having special health care needs if the respondent indicated that the child had any of these conditions.

We used descriptive statistics and bivariate analyses to calculate frequencies, proportions, and unadjusted associations for each variable. We compared the characteristics of children in rural and urban areas by using Pearson χ^2^ and Mantel–Haenszel tests as appropriate. We analyzed data in terms of the child, per NSCH guidelines (childhealthdata.org). We used multivariable logistic regression to run 3 separate models to examine the relationship between 1) rurality and food insecurity, 2) rurality and physical inactivity, and 3) rurality and overweight or obesity. To ensure accurate model estimates, we used the appropriate survey sampling weights, clusters, and strata used by the NSCH in all analyses. We conducted all analyses in SAS version 9.4 (SAS Institute Inc). This study was approved as exempt by the University of South Carolina institutional review board.

## Results

Less than one-tenth (8.3%) of the children in our sample resided in a rural area, three-quarters (75.5%) were aged 10 to 15 years, and just over one-half (51.2%) were boys (Table 1). Just under one-half were non-Hispanic White (48.6%), more than one-quarter were Hispanic (27.0%), and 14.2% were non-Hispanic Black. Nearly one-quarter (24.8%) of the sample had special health care needs. The primary language in the household was not English for 15.2% of respondents. Most (69.1%) of the children in the sample had caregivers with some college education or more, and most (62.0%) had 2 parents currently married. Less than one-fifth (17.3%) of the sample were living below the FPL.

**Table 1:** Characteristics of Children and Households Reported by Respondents in the National Survey of Children’s Health in 2019 and 2020, Overall (N = 23,199) and by Rural–Urban Residence

Characteristic	All, %	Rural, %	Urban, %	*P* value^a^
**Overall**	100.0	8.3	91.7	—
**Child**				
Sex				
Male	51.2	52.2	51.1	.57
Female	48.8	47.8	48.9	
Age, y				
10–12	37.6	37.9	37.5	.85
13–15	37.9	37.8	38.4	
16–17	24.6	24.6	23.7	
Race and ethnicity				
Hispanic	27.0	8.5	28.6	<.001
Non-Hispanic Black	14.2	8.6	14.7	
Non-Hispanic White	48.6	77.8	45.9	
Non-Hispanic Other^b^	10.2	5.1	10.7	
Has special health care needs	24.8	24.8	24.8	.98
**Caregiver or household**				
Primary language spoken in household is not English	15.2	2.9	16.3	<.001
Highest level of educational attainment of caregiver in the household				
High school diploma or less/GED	30.9	38.0	30.3	<.001
Some college or more	69.1	62.0	69.7	
Family structure				
2 Parents, currently married	62.0	61.3	62.1	.04
2 Parents, not currently married	6.9	8.3	6.8	
Single caregiver household	22.9	20.2	23.1	
Other	8.2	10.2	8.0	
Household income as percentage of federal poverty level				
0–99	17.3	20.8	16.9	<.001
100–199	22.9	26.9	22.5	
200–399	29.4	34.0	29.0	
≥400	30.5	18.3	31.6	

Abbreviations: — , does not apply; GED, General Educational Development.

a *P* values were calculated to compare rural–urban differences in respondent characteristics by using Pearson χ^2^ tests and Mantel–Haenszel tests.

b Includes non-Hispanic Asian, American Indian or Alaska Native, Native Hawaiian or other Pacific Islander, multiracial, and biracial.

### Rural–urban differences in characteristics of children and caregivers/households

Compared with children residing in urban areas, children residing in rural areas were more likely to be non-Hispanic White (77.8% vs 45.9%; *P* < .001) (Table 1). English was not the primary language spoken in the household for a smaller portion of rural children than urban children of (2.9% vs 16.3%; *P* < .001). A larger proportion of rural children than urban children had a caregiver with a high school diploma or GED or less (38.0% vs 30.3%; *P* < .001). Rural children were more likely than urban children to live below the poverty level (20.8% vs 16.9%; *P* < .001).

### Rural–urban differences in weight, food security, and physical activity

In bivariate analyses, rural children were more likely than urban children to be overweight or obese (37.6% vs 32.1%; *P* = .003) (Table 2). A smaller percentage of rural children than urban children were physically inactive (79.3% vs 84.0%; *P* = .005). Compared with their urban counterparts, rural children were more likely to have food insecurity (39.6% vs 31.1%; *P* < .001), both for mild food insecurity (34.0% vs 26.3%; *P* < .001) and moderate to severe food insecurity (5.7% vs 4.9%; *P* < .001).

**Table 2:** BMI Category, Physical Activity, and Food Security Reported by Respondents to the 2019–2020 National Survey of Children’s Health, Overall (N = 23,199) and by Rural–Urban Residence

Characteristic	All, %	Rural, %	Urban, %	*P* value^b^
**BMI^a^**				
Underweight	6.1	6.6	6.0	.003
Healthy weight	61.4	55.8	61.9	
Overweight or obese	32.5	37.6	32.1	
**Physical activity**				
Inactive (less than every day of the week)	83.6	79.3	84.0	.005
Active (60 min of physical activity every day)	16.4	20.7	16.0	
**Food security**				
Food secure	68.1	60.4	68.9	<.001
Food insecurity	31.9	39.6	31.1	
Mild food insecurity	26.9	34.0	26.3	
Moderate to severe food insecurity	4.9	5.7	4.9	

Abbreviation: BMI, body mass index.

a BMI is calculated for children aged 10 to 17 years as 1) underweight (BMI <5th percentile), 2) healthy weight (5th percentile to <85th percentile), and 3) overweight or obese (≥85th percentile). These categories were established by the Centers for Disease Control and Prevention (22).

b *P* values were calculated to compare rural–urban differences in respondent characteristics by using Pearson χ^2^ tests and Mantel–Haenszel tests.

In multivariable logistic regression analysis, rural children had higher odds of being overweight or obese than urban children (adjusted odds ratio [AOR] = 1.30; 95% CI, 1.11–1.52) (Table 3). Other significant covariates for overweight or obesity included the child’s race and ethnicity, sex, age, and special health care needs, educational attainment of the caregiver, and poverty level. Non-Hispanic Black children had a higher likelihood than non-Hispanic White children of being overweight or obese (AOR = 1.54; 95% CI, 1.28–1.85). Children with special health care needs had higher odds of overweight or obesity than children who did not have special health care needs (AOR = 1.26; 95% CI, 1.11–1.43). Children living below 400% of the FPL had a higher likelihood of overweight or obesity than children living at 400% or more above the FPL.

**Table 3:** Prediction of Overweight or Obesity, Physical Inactivity, and Food Security by Rural–Urban Residence Reported by Respondents (N = 23,199) to the 2019–2020 National Survey of Children’s Health^a^

Characteristic	Model 1: Overweight or obesity	Model 2: Physical inactivity	Model 3: Food insecurity
**Rurality**			
Rural	1.30 (1.11–1.52)^b^	0.92 (0.75–1.12)	1.18 (0.99–1.40)
Urban	1 [Reference]	1 [Reference]	1 [Reference]
**Child**			
Race and ethnicity			
Hispanic	1.50 (1.28–1.85)^b^	1.70 (1.35–2.15)^b^	1.26 (1.04–1.53)^b^
Non-Hispanic Black	1.54 (1.28–1.85)^b^	1.41 (1.12–1.78)^b^	1.13 (0.92–1.38)
Non-Hispanic White	1 [Reference]	1 [Reference]	1 [Reference]
Non-Hispanic Other^c^	0.94 (0.79–1.12)	1.13 (0.91–1.41)	1.10 (0.90–1.34)
Sex			
Male	1 [Reference]	1 [Reference]	1 [Reference]
Female	0.80 (0.71–0.91)^b^	1.62 (1.41–1.87)^b^	1.06 (0.93–1.22)
Age, y			
10–12	1.28 (1.11–1.48)^b^	0.74 (0.63–0.86)^b^	0.99 (0.85–1.17)
13–15	1 [Reference]	1 [Reference]	1 [Reference]
16–17	0.94 (0.80–1.10)	1.04 (0.87–1.24)	0.90 (0.76–1.07)
Has special health care needs	1.26 (1.11–1.43)^b^	1.25 (1.07–1.47)^b^	1.38 (1.20–1.59)^b^
Primary language spoken in household is not English	1.00 (0.78–1.29)	1.63 (1.17–2.29)^b^	0.58 (0.45–0.76)^b^
**Caregiver or household**			
Education of caregiver			
High school diploma or less/GED	1.28 (1.09–1.51)^b^	0.87 (0.72–1.05)	1.05 (0.88–1.24)
Some college or more	1 [Reference]	1 [Reference]	1 [Reference]
Family structure			
2 Parents, currently married	1 [Reference]	1 [Reference]	1 [Reference]
2 Parents, not currently married	1.09 (0.83–1.43)	0.97 (0.71–1.33)	1.63 (1.23–2.14)^b^
Single caregiver household	1.15 (0.98–1.34)	1.11 (0.93–1.34)	1.53 (1.29–1.80)^b^
Other	0.91 (0.64–1.31)	0.80 (0.53–1.21)	1.02 (0.68–1.53)
Household income as percentage of federal poverty level			
0–99	1.27 (1.01–1.59)^b^	0.71 (0.56–1.02)	4.88 (3.84–6.19)^b^
100–199	1.59 (1.33–1.92)^b^	0.90 (0.73–1.12)	5.27 (4.30–6.46)^b^
200–399	1.34 (1.17–1.55)^b^	1.04 (0.86–1.21)	3.48 (2.92–4.14)^b^
≥400	1 [Reference]	1 [Reference]	1 [Reference]

a All values are adjusted odds ratio (95% CI), determined by Wald test.

b Significant at *P* < .05.

c Includes non-Hispanic Asian, American Indian or Alaska Native, Native Hawaiian or other Pacific Islander, multiracial, and biracial.

We found no significant associations between rurality and physical inactivity or food insecurity (Table 3). Non-Hispanic Black children had higher odds of physical inactivity than non-Hispanic White children (AOR = 1.41; 95% CI, 1.12–1.78). Girls had a higher likelihood of physical inactivity than boys (AOR = 1.62; 95% CI, 1.41–1.87). Children with special health care needs had a higher likelihood of physical inactivity than children who did not have special health care needs (AOR = 1.25; 95% CI, 1.07–1.47). Children who lived in households where English was not the primary language spoken had higher odds of physical inactivity than children who lived in households where English was the primary language spoken (AOR = 1.63; 95% CI, 1.17–2.29).

In our analysis of food insecurity, Hispanic children had higher odds of being food insecure than their non-Hispanic White counterparts (AOR 1.26; 95% CI, 1.04–1.53) (Table 3). Children with special health care needs had higher odds of food insecurity than children without special health care needs (AOR 1.38; 95% CI, 1.20–1.59). Children residing in a single caregiver household had a higher likelihood of food insecurity than children residing with 2 parents, currently married (AOR 1.53; 95% CI, 1.29–1.80). Children living below 400% of the FPL had a higher likelihood of being food insecure than children living at 400% or more above the FPL. Households in which English was not the primary language spoken were less likely to be food insecure than households in which the primary language spoken was English.

## Discussion

This study examined rural–urban differences in overweight or obesity, food security, and physical inactivity among a national sample of US children. Weight status, nutrition, and physical activity are among the leading health priorities in rural areas (26). We found that although rural children had higher odds of being overweight or obese than urban children, associations between rurality and physical inactivity and food insecurity were not significant.

Our finding that non-Hispanic Black children had higher odds of being overweight or obese is consistent with several studies in which similar findings were reported (7,27,28). Other factors, including racial segregation and crime, have been associated with a higher obesity prevalence (28), which may at least partially explain the increased odds of overweight or obesity among racial and ethnic minority children in our study, despite adjusting for sociodemographic factors. Other research showed that stress may play an important role in obesity among those with food insecurity; among a largely White sample of children, adolescents, and young adults residing in rural upstate New York, McClain et al found that household food insufficiency was not associated with BMI trajectory (29). However, increasing maternal stress was associated with a worsening BMI trajectory among those in food-insufficient households compared with food-sufficient households (29).

We found that proportionately more rural children than urban children had mild-moderate food insecurity but that the association between rurality and food insecurity was not significant in the adjusted model. The unadjusted differences in food insecurity between rural and urban children appeared to be mostly mitigated by Hispanic ethnicity, special health needs, and household income levels. Recent research suggested that rates of fruit and vegetable consumption were lower among rural adolescents than among suburban and urban adolescents but that proportionately fewer rural adolescents than suburban adolescents consumed junk foods, sugar-sweetened beverages, and sugary foods (27). However, adjusting for sex, race and ethnicity, and family household income showed that consumption of fruits and vegetables and these unhealthy foods was higher only among suburban adolescents (27). These findings suggest that income differences by rurality is an important factor that may underlie rural–urban differences in food insecurity and dietary behavior.

Although a smaller percentage of rural children were physically inactive compared with urban children, we found no rural–urban differences after adjusting for the sociodemographic characteristics of the child and the child’s household and the child’s special health needs status. This finding is similar to recent research indicating that levels of moderate to vigorous physical activity do not differ by rurality (27). Previous research showed that the prevalence of physical inactivity is lower among rural children than urban children aged 2 to 11 years but is similar among those aged 12 to 19 years (30). Poverty appears to be a big driver of rural–urban differences in food insecurity, and policies to reduce poverty and promote economic prosperity are needed in rural areas to address food insecurity.

### Strengths and limitations

This study is the most up-to-date examination of rural–urban differences in weight status, physical activity, and food security among a large sample of children. It has several potential limitations. First, the data may be subject to recall and social desirability bias; clinical measurements of height and weight, physical activity, and food security would be more accurate than the recollections of survey respondents. Second, the category of physically inactive may have included children who might have been active but not sufficiently active to meet our criteria for physically active (ie, 60 minutes every day). Third, the NSCH uses an address-based sampling process, which limits the sample to children residing at a physical address (eg, no children experiencing homelessness were included). Fourth, because the NSCH suppresses residential data for some states, the generalizability of our study results is limited. Future research should use more nuanced classifications of rurality and data that represent states that are majority rural. Fifth, use of the NSCH geographic variable for determining rurality may mask differences in obesity, physical inactivity, and food insecurity that might be unmasked by using other definitions of rurality. A recent study showed that rural–urban inequities in childhood obesogenic environments varied according to the definition of rural used (31). We hypothesized that obesogenic environments would vary across levels of rurality. Sixth, some of the data collection for this study occurred during the COVID-19 pandemic (July 2020 through January 2021). This factor may limit the generalizability of our study results; because some children may have been primarily at home instead of school, they may have been engaging in more or less physical activity during the COVID-19 pandemic than during non–COVID-19 times.

### Conclusions

Information from this study is timely for policy makers and community partners to make reasonable decisions on the allocation of healthy weight and obesity prevention programs for children in rural settings, as the study elucidates rural–urban differences in overweight or obesity. It provides information for public health programming goals and appropriate dietary and physical activity interventions that are needed to reduce disparities in obesity prevention among children. Further longitudinal research is needed to clarify the pathways between physical activity, food insecurity, and obesity among rural children, compared with urban children, and examine diversity in physical activity, food insecurity, and obesity in rural areas.
